# Relationship of finger dermatoglyphics with ameloglyphics and their values as dental caries predictors in primary teeth

**DOI:** 10.1186/s12903-025-07027-6

**Published:** 2025-10-14

**Authors:** Lamya Mohammed Grawish, Basma El-Sayed Hamza, Youssry M. El-Hawary, Nasr Mohamed Attia

**Affiliations:** 1 Ministry of Health and Population, Mansoura, Egypt; 2 Dental Public Health and Preventive Dentistry, Faculty of Dentistry, Mansoura University, Mansoura, Egypt; 3 Oral Biology, Faculty of Dentistry, Mansoura University, Mansoura, Egypt; 4 Dental Public Health and Preventive Dentistry, Faculty of Dentistry, Mansoura University, Mansoura, Egypt

**Keywords:** Dermatoglyphics, Ameloglyphics, Enamel lamellae, Enamel tufts, Enamel spindles, Ca, P, C and O weight content

## Abstract

**Background:**

In the clinical setting, it would be useful to predict which children are at the highest risk of caries to take effective preventive measures. This study aims to evaluate the relationship between finger dermatoglyphics and ameloglyphics and to predict caries susceptibility of primary teeth in association with finger dermatoglyphics.

**Methods:**

Finger dermatoglyphics and ameloglyphics were recorded using the stamp ink pad and cellophane tape techniques, respectively. The association between finger dermatoglyphics and ameloglyphics was analyzed. The hypocalcified areas, micromorphological patterns and the microchemical weight contents of the enamel were investigated using a light microscope, a scanning electron microscope, and energy-dispersive X-ray spectroscopy.

**Results:**

There was a significant association between the fingerprint dermatoglyphics and ameloglyphics (Fisher’s exact test = 29.503, *P* = 0.000) and, this association was a moderate one (Cramér’s V = 0.385). The hypocalcified areas of enamel in the form of lamellae, tufts, and spindles were predominated in the teeth related to the arch fingerprint and less predominated in the teeth related to the loop fingerprint patterns. Micromorphological investigation showed enamel rods of irregular and uneven thickness in the teeth related to the arch fingerprint pattern. Whereas, enamel rods were of regular and even thickness in the teeth related to the loop fingerprint patterns. Microchemical analysis for the weight content (kα) of Ca, P, C, and, O elements in the enamel of the teeth related to the arch, loop and whorl fingerprints patterns revealed that the mean values and standard deviations were 23.53 ± 0.15, 25.72 ± 0.02 and 24.77 ± 0.02 for Ca; 12.20 ± 0.05, 13.02 ± 0.01 and 12.17 ± 0.01 for P; 29.47 ± 0.02, 28.77 ± 0.02 and 29.24 ± 0.02 for C; and 34.51 ± 0.01, 32.36 ± 0.02 and 33.68 ± 0.02 for O, respectively. One-way ANOVA statistical test revealed significant differences for the Ca, P, C and O weight content (kα) between three groups (*P* value = 0.000).

**Conclusion:**

There is a moderate association between finger dermatoglyphics and ameloglyphics, and finger dermatoglyphics could be used as a handy tool for predicting carious lesions in primary teeth.

## Background

Dermatoglyphics is the study of epidermal ridge patterns and lines on the soles, toes, palms, and fingers, a term coined by the anatomist Harold Cummins in 1926 [1]. Dermatoglyphics has been studied in various malformations caused by autosomal aberrations, sex chromosomal aberrations, inherited or genetic malformations, and systemic disorders [2–5]. In dentistry, dermatoglyphics has been studied in dental and skeletal malocclusions, dental caries, periodontal diseases, hereditary gingival fibromatosis, cleft lip and palate, and potentially malignant disorders of the oral cavity [6–10]. Dermatoglyphics was a simple, inexpensive, bedside diagnostic aid for chromosomal aberrations and various heritable diseases [11].

Fingerprints are epidermal structures made by the papillary ridges that are formed in early embryonic life, during the 3rd to 4th month of fetal life, and they remain permanent during the whole life, except for dimensions in proportion to the growth of an individual [12]. Normal or abnormal genetic messages carried in the genome are decoded and translated during this period and these processes are reflected in dermatoglyphics [13]. Many structures of the body develop during the same period as the finger ridges, abnormal developmental insults on these structures are likely to be reflected in the dermatoglyphic patterns [14].

A fingerprint is divided into three broad categories: the whorl, which is reminiscent of a whirlpool, the loop, which is teardrop- shaped, and the arch, which looks a bit like a cross-section of a hill [15]. There may be two subpatterns of arches: plain or tented; two subpatterns of loops, which may be ulnar or radial depending upon the direction they face and, four subpatterns of whorls: plain, central pocket loop, double loop, and accidental patterns [16].

There has been a growing interest in studying enamel rod end patterns. These patterns are termed toothprints, and the study of these prints is known as ameloglyphics. They are considered a hard tissue analog to fingerprints [17]. Enamel is a unique tissue in that once formed, it is not remodeled, as mature enamel does not contain any synthetic cells. Enamel rod morphology correlates with the morphology of ameloblasts and, alterations to the enamel matrix are reflected as defects in the structural organization of enamel [18].

The enamel rod is narrowest at its origin and gradually enlarges as it approaches the outer enamel surface, with an average diameter of 4.0 μm at the dentin side. Cross striations and undulations segment the enamel along its length. The rods take up key-hole or fish-scale patterns in cross-sections and appear cylindrical in longitudinal Sect [19]. The head, which is called the rod core, is oriented toward the tooth’s crown whereas, the tail, which is called the rod sheath, is oriented toward the tooth’s cervical margin. The rod core has tightly packed hydroxyapatite crystals, whereas the rod sheath has its crystals less tightly packed and has more space for organic components. These rod structures can usually be visualized within ground sections and/or with the use of a scanning electron microscope (SEM) on enamel that has been acid etched [20].

A variation in dermatoglyphics was found between the group of early childhood caries (ECC) and caries-free subjects, indicating that dermatoglyphic patterns could be used as a non-invasive predictive tool for children with ECC [21]. Therefore dermatoglyphics holds an importance in this area as a future caries predictor [22]. Dermatoglyphic is an economical tool for the preliminary diagnosis of diseases of suspected genetic origin like, dental caries [23]. In addition, a relationship was found between fingerprint pattern and enamel mineral content of the primary teeth as the phosphorus mineral content was the highest in the arch pattern while calcium one was the highest in the loop pattern [24]. Regarding these findings, a research question was developed which, is there is a relationship between finger dermatoglyphics and ameloglyphics and if finger dermatoglyphics could be used as a predicator for caries susptability in primary teeth. Our null hypothesis was that no relation between finger dermatoglyphics and ameloglyphics and no relation between finger dermatoglyphics and the prediction of dental caries.

## Methods

### Sample size calculation, subjects, and sampling technique

The sample size was statistically determined using G* Power 3.1.9.2 software. It was fundamentally dependent on our research question, study design and statistical analysis. The test family was F test, and the statistical test was ANOVA: fixed effects, omnibus, one-way. The type of power analysis was A priori: Compute the required sample size given α, power, and effect size. The input parameters were an error probability (α) of 0.05, an effect size (f) of 0.40, a power of 0.95, and the number of groups was 3. Choosing an effect size of 0.40 for an ANOVA was dependent on previous studies that suggests a substantial difference or relationship between the groups being compared [9, 11, 21]. The estimated sample size was 102 (34 teeth/group). One hundred and two child patients were consecutively selected based on predefined inclusion and exclusion criteria to determine who was eligible and ineligible for the participation in the study. Therefore, 34 child patients were selected based on each fingerprint pattern (arch, loop, and whorl).

This study was designed to evaluate the relationship between finger dermatoglyphics and ameloglyphics and to predict caries susceptibility of primary teeth with finger dermatoglyphics. The upper right or left primary 2nd molars were extracted and examined to obtain the tooth print pattern for each child. Thus, the total number of teeth examined was identical to the number of the child patients involved in the study (total = 102, 34 teeth per each group). After obtaining the toothprint patterns and to determine which group of teeth were more susceptible to the dental caries, the teeth were processed to get specimens for investigating the hypocalcified areas of the enamel including enamel lamellae, tufts and spindles, exploring the micromorphological pattern of the enamel including rods, interrods and rod sheaths and assessing the microchemical elements of Ca, P, C and O. All selected teeth were included and processed for examination. Convenience sampling was used for micromorphological investigation and microchemical analysis of the enamel (*n* = 10). Convenience sampling approach was dependent on the fact that it is quick and cost-effective [25]. Ten specimens from each group were selected randomly from the predefined list of the participant. Research randomizer software (https://www.randomizer.org/) was used for simple randomization.

### Inclusion and exclusion criteria

The inclusion and exclusion criteria were based on the research question, which was determined before the study begins. The purposes were to ensure homogeneity, reduce bias and improve efficiency [26]. The inclusion criteria were (1) boys and girls in the age range of 10 to 12 years, (2) children that have undergone extraction of their upper primary 2nd molars, (3) the reasons for extraction were exfoliation, periapical pathosis, and failure of endodontic treatment and, (4) decayed or restored molars with their buccal surfaces are intact and in the same time their mesial or distal halves are also intact. Whereas the exclusion ones were (1) badly destructed upper primary 2nd molars, (2) history of preventive measures of dental caries, (3) children with Down syndrome, Turner syndrome, and cerebral palsy, and (4) children with adermatoglyphia, and children with developmental disturbances of genetic and chromosomal disorders occurring during ridge differentiation.

### Ethical considerations and approvals

This study was carried out at three centers from September 2024 to August 2025. The protocol was reviewed and approved by the institutional review boards (M01010024PP) and independent ethics committees of the participating dental centers and adhered to the World Medical Association Declaration of Helsinki. Consecutive child patients were recruited from the Department of Pediatric Dentistry and Dental Public Health, Faculty of Dentistry, Mansoura University; the Pediatric Dental Department, Mansoura International Hospital; and the Outpatient Dental Clinic, Aga Central Hospital. Informed consent was obtained from the child’s parents to participate in the study. This study is evaluated based on the standard reporting of caries detection and diagnostic studies (STARCARDDS) to enhance the transparency and accuracy of reporting [27].

### Inter- and Intra-examiner calibration

Regarding the captured images for the fingerprint patterns, the toothprint patterns, the hypocalcified areas of enamel and, the micromorphological structure of enamel, the biologist supervisor trained the candidate who conducted the study to examine the fingerprint and toothprint patterns and to rate the number and the extent of the hypocalcified areas of enamel, including lamellae, tufts, spindles and, in addition to rate the thickness and the course of the rods, interrods and rod sheaths of the enamel. Thus, a total of two examiners were involved (YH and LG). A simple percent agreement calculation was used for intra-examiner and inter-examiner agreements. Percentage agreement and the kappa statistic (κ) were used to measure agreement at 80% power and an alpha level of 0.05. The study standards required close agreement for the intra-examiner and inter-examiner variability. A range that falls within 75% to 90% is considered an acceptable percentage of agreement.

### Obtaining fingerprint patterns

The fingerprint patterns were recorded from the left thumb of the male child or the right thumb of the female child according to the method described by Belludi et al. (2021). The fingerprint patterns were analyzed with a magnifying glass with 3× power (Handheld Magnifying Glass 3X Power, China). The dermatoglyphic analysis comprised the following: qualitative analysis, including the patterns of fingerprints, i.e., loops, whorls, and arches [6].

### Determining toothprint patterns

The teethprint patterns were recorded according to the method described by Qin et al. (2023) [28]. The photomicrographs were subjected to biometric analysis using RapidResizer software (https://online.rapidresizer.com/start.php?plugin=start).

### Determining the hypocalcified structures of enamel

Ground sections were prepared to examine the hypocalcified structures of the enamel, which are enamel lamellae, tufts, and spindles. The root side of each tooth was mounted on acrylic resin to be sectioned using metallographic PICO 155 precision saw (PACE Technology, USA). At first, the sound part, whether the distal or mesial side, was separated from the decayed portion to get our specimens. Then, starting from the cusp tips till about two-thirds of the crown, each specimen was cut buccolingually into two slabs. One slab of 0.30 mm thickness is to be used for preparing a longitudinal ground section to examine the presence or absence of enamel spindles. The second longitudinal slab was cut to a thickness of 2 mm, and it was used for SEM and EDX examination. The remaining one-third of the crown portion was cut transversely to get a third slab of 0.30 mm thickness to be used for preparing a transverse ground section to examine the presence or absence of enamel lamellae and tufts.

After sectioning, 250 μm thick ground sections were made by using Arkansas stone. The thickness of each section was confirmed using a digital vernier caliper. The grounded sections were cleared in xylene for one minute and then they were dried, sandwiched between a microscope slide and a coverslip with the Canada balsam as a mounting medium to be investigated with the light microscope [29] (Olympus^®^, CX22, Japan) attached to a digital camera with 0.5 photo adaptor (ToupCam^®^, model no. XCAM1080PHA) using 20x objective. In each section, three fields were investigated, and the mean values for the three fields were considered as one reading for each structure. The types of enamel lamellae, whether type A, B or C, the extension of the enamel tufts whether 1/3 or 1/5, were identified, and their numbers were counted for each specimen, and the length of the enamel spindles, whether short, medium or long.

### Micromorphological investigation and microchemical analysis of the enamel

The specimens were accurately cut buccolingually into sections of 2 mm thickness from the intact mesial or distal halves using the metallographic PICO 155 precision saw (PACE Technology, USA) to prepare the specimens (*n* = 10/group) for micromorphological and microchemical analyses using SEM and EDX. Element mapping was performed to detect the difference in content of Ca, P, C, and O between the teeth related to the arch, loop and whorl patterns.

### Statistical analyses

All the raw data were subjected to statistical analyses using the Statistical Package for the Social Sciences software, version 26. The distribution of all variables was examined for normality using Shapiro-Wilk normality testing. The categorical data of the fingerprint and toothprint patterns were analyzed using the frequency and percentage distribution. Fisher’s exact test was used to detect the association between fingerprint and toothprint patterns followed by Cramér’s V to determine the degree of association, the significance level was set at *P* ≤ 0.05. Independent-samples Kruskal-Wallis test was used to detect the total significant difference between the hypocalcified structures among the teeth related to the fingerprint patterns followed by Mann-Whitney U test for pairwise comparison, the significance level was set at *P* ≤ 0.05. Whereas, the continuous data of Ca, P, C and O weight content (kα) were compared using one-way ANOVA, followed by an LSD post-hoc test, the significance level was set at *P* ≤ 0.05.

## Results

The Cohen’s Kappa statistical test showed strong intra-examiner and inter-examiner reliability (k = 0.87).

### Fingerprint patterns

It was found that among the arch, loop, and whorl basic fingerprint patterns, there were subpatterns in the form of plain arch, tented arch, ulnar loop, radial loop, plain whorl, central pocket loop, double loop, and accidental. The valid percentage for the plain and tented arch subpatterns was 79.40% and 20.60%, respectively. Whereas the ulnar and radial loop subpatterns were 88.20% and 11.80%, respectively and for the plain whorl, central pocket loop, double loop and accidental subpatterns were 82.40%, 8.80%, 5.90% and 2.90%, respectively (Table 1).

**Table 1:** Frequency and percentage of plain, tented, ulnar, radial, plain whorl, central pocket loop, double loop and accidental subpatterns of the arch, loop and whorl fingerprint patterns

	Fingerprint	Frequency	Percent	Valid Percent	Cumulative Percent
Valid	Plain	27	26.50%	79.40%	79.40%
	Tented	7	6.90%	20.60%	100.0%
	Total	34	33.30%	100.0%	
	Ulnar	30	29.40%	88.20%	88.20%
	Radial	4	3.90%	11.80%	100.0%
	Total	34	33.30%	100.0%	
	Plain	28	27.50%	82.40%	82.40%
	Central pocket loop	3	2.90%	8.80%	91.20%
	Double loop	2	2.0%	5.90%	97.10%
	Accidental	1	1.0%	2.90%	100.0%
	Total	34	33.30%	100.0%	

### Toothprint patterns

**Fig. 1:**
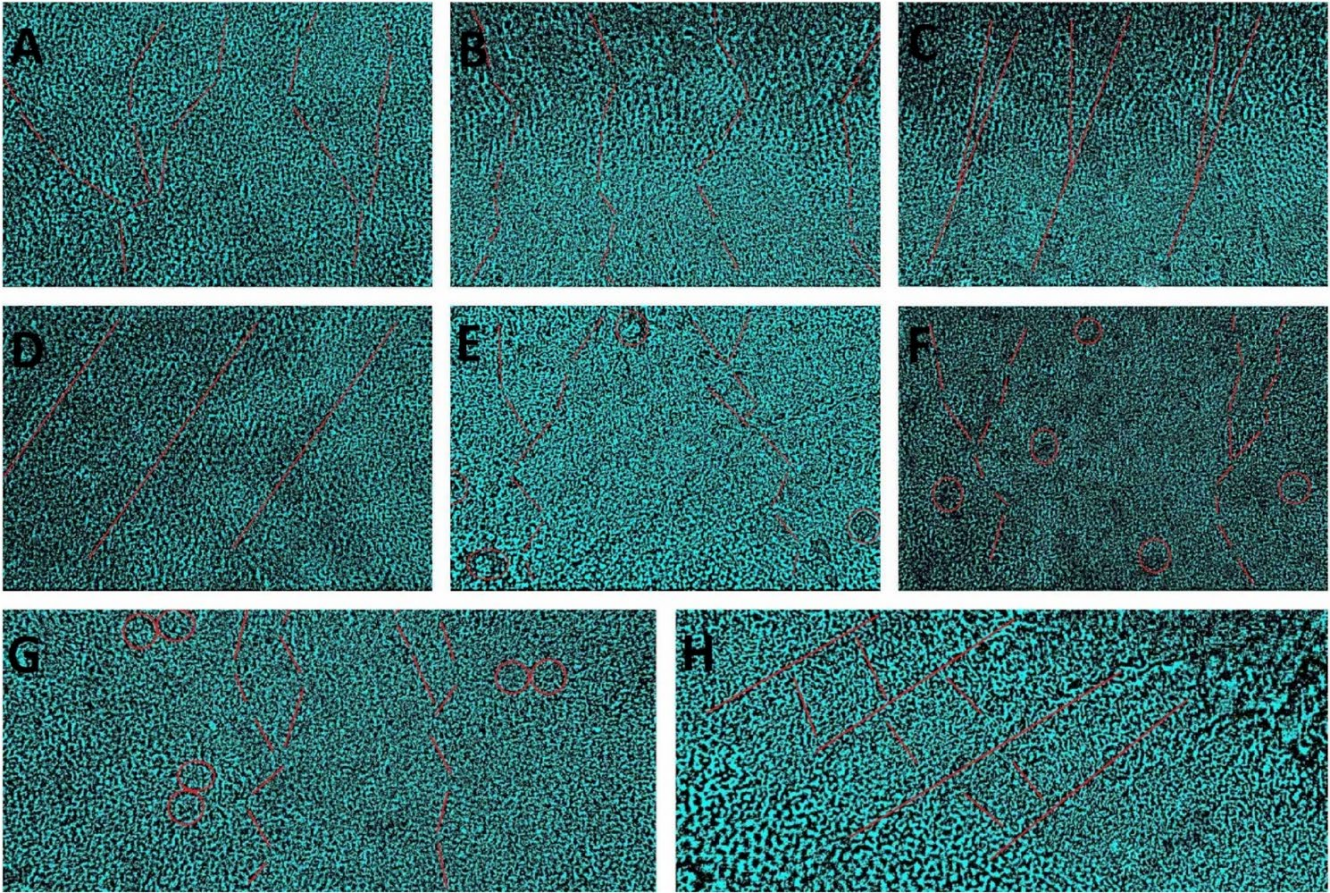
Photomicrographs shows biometric conversion of ameloglyphic patterns using RapidResizer software; wavy branched (**A**), wavy unbranched (**B**), linear branched (**C**), linear unbranched (**D**), wavy branched with whorl open (**E**), wavy branched with whorl closed (**F**), wavy branched with loop pattern (**G**), linear unbranched with stem like (**H**)

**Fig. 2:**
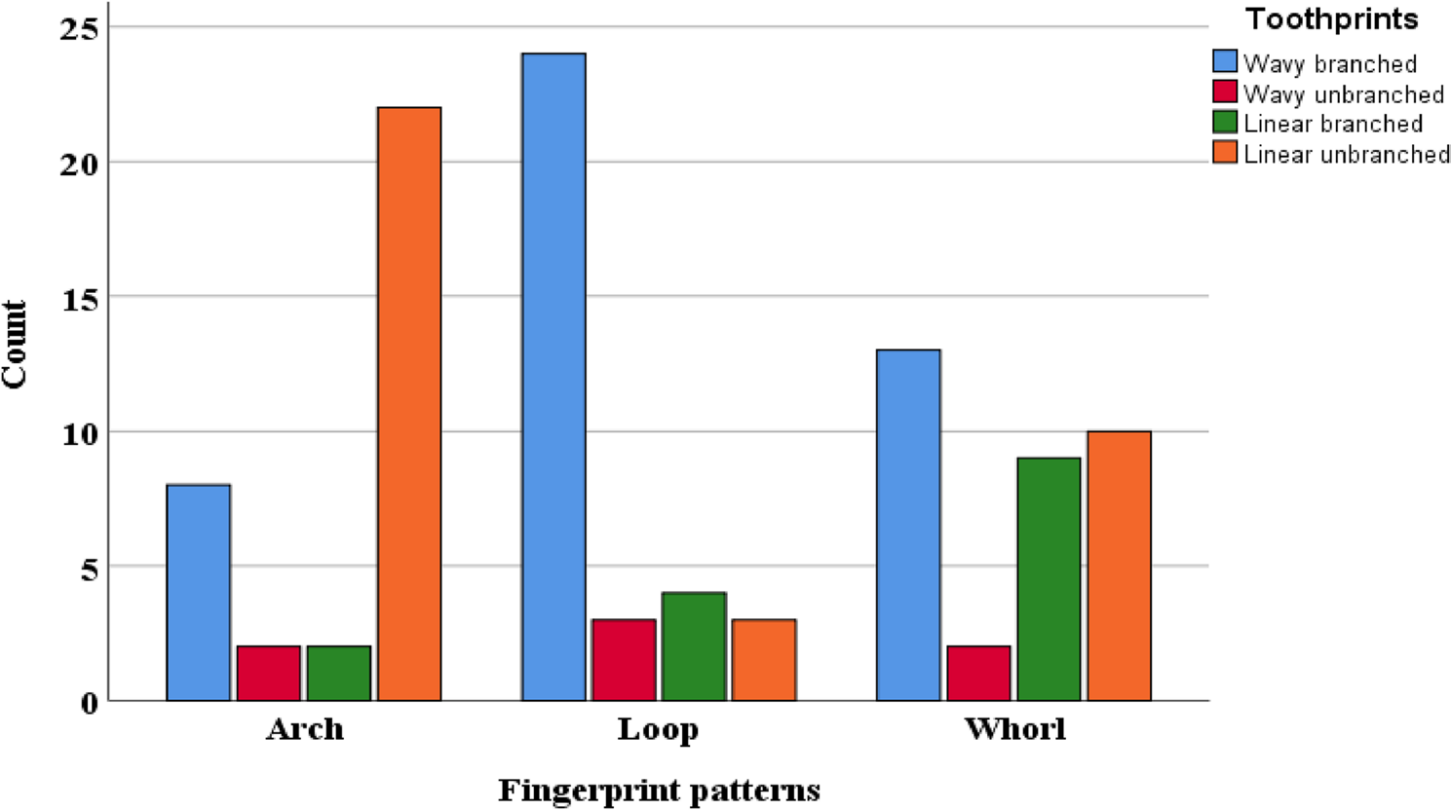
Clustered bar chart for the frequencies of toothprint patterns among arch, loop, and whorl fingerprint patterns

There were major subpatterns for the toothprints. The predominated subpatterns were wavy branched, wavy unbranched, linear branched, and linear unbranched. Whorl open, whorl closed, loop pattern, and stem-like were seen only in combination with the predominant major subpatterns (Fig. 1). Regarding arch fingerprint, the percentage of toothprints for the wavy branched, wavy unbranched, linear branched, and linear unbranched were 23.50%, 5.90%, 5.90% and 64.70%, respectively. Meanwhile, the same toothprints for the loop fingerprint were 70.60%, 8.80%, 11.80%, and 8.80%, respectively, and for the whorl fingerprint were 38.20%, 5.90%, 26.50%, and 29.40%, respectively (Fig. 2; Table 2).

**Table 2:** Frequency and percentage of wavy branched, wavy unbranched, linear branched and linear unbranched related to the arch, loop and whorl fingerprint patterns

Fingerprint	Frequency (percentage)			
	Wavy branched	Wavy unbranched	Linear branched	Linear unbranched
Arch	8 (23.50%)	2 (5.90%)	2 (5.90%)	22 (64.70%)
Loop	24 (70.60%)	3 (8.80%)	4 (11.80%)	3 (8.80%)
Whorl	13 (38.20%)	2 (5.90%)	9 (26.50%)	10 (29.40%)

### Association of fingerprint patterns with toothprint patterns

The value for Fisher’s exact test was 29.503, and the significance was 0.000. This means that there was a highly significant difference between observed data and expected data. There was an association between the fingerprints and toothprints categorical variables and this association was not due to chance (Table 3). To measure how strongly the fingerprints were associated with the toothprints, Cramér’s V statistical test was used. Cramér’s V is used when there is more than a 2 × 2 contingency. The value of the Cramér’s V was 0.385, indicating a moderate correlation between fingerprints and toothprints categorical variables. The level of significance for the Cramér’s V test was 0.000, indicating a highly significant difference for this correlation (Table 4).

**Table 3:** Chi-Square statistical test for the association of fingerprint patterns with the toothprint patterns

	Value	df	*P* value (2-sided)	Exact *P* value (2-sided)	Exact *P* value (1-sided)	Point Probability
Pearson Chi-Square	30.248^a^	6	0.000	0.000		
Likelihood ratio	30.864	6	0.000	0.000		
Fisher’s exact test	29.503			0.000		
Linear-by-linear association	3.900^b^	1	0.048	0.053	0.027	0.005
*N* of valid cases	102					

**Table 4:** Correlation between fingerprint patterns with the toothprint patterns

		Value	Approximate *P* value	Exact *P* value
Nominal by Nominal	Phi	0.545	0.000	0.000
	Cramer’s V	0.385	0.000	0.000
*N* of Valid Cases		102		

### Hypocalcified areas

**Fig. 3:**
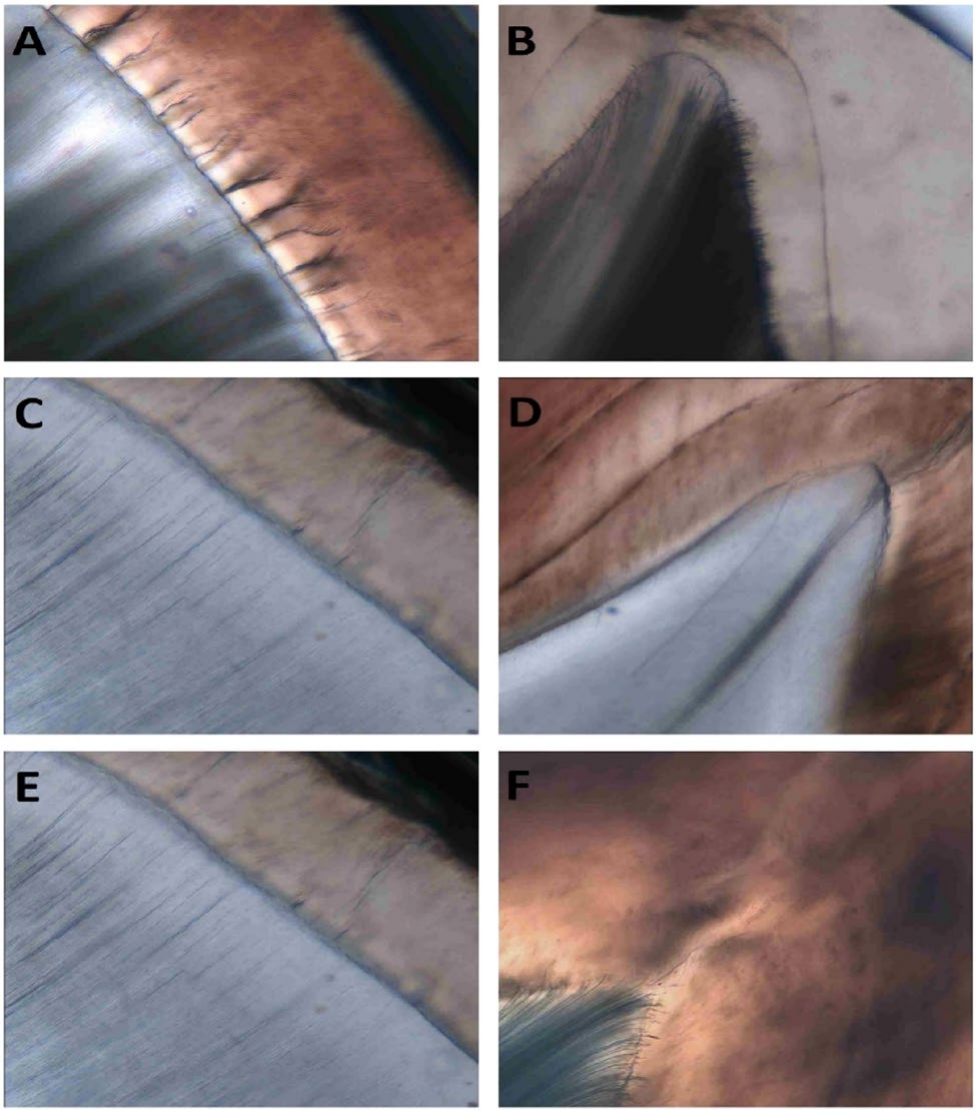
Ground sections of the upper primary 2nd molar showing enamel lamellae, tufts and spindle. The enamel lamellae, tufts, and spindles are presented with a greater number in the teeth related to the arch fingerprint patterns (**A**, **B**); whereas in the loop fingerprint patterns, the ground sections show the fewer number of the same structures (**C**, **D**). The ground sections of the teeth related to the whorl fingerprint patterns have the same structures with a higher number than that presented in the loop fingerprint patterns but less than that found in the arch fingerprint patterns (**E**, **F**). A, C and E are cross sections where B, D, and F are longitudinal sections (magnification, 20X)

Enamel lamellae and tufts were found in the cross sections, and enamel spindles were found in the longitudinal ones (Fig. 3). The frequency and the valid percentages for the hypocalcified structures in the enamel are presented in Table 5. Independent-samples Kruskal-Wallis statistical test for the results of the types of enamel lamellae, tufts, and spindles among the teeth related to the arch, loop, and whorl fingerprint patterns revealed a highly significant difference between lamellae types of the three fingerprint patterns (*P* value = 0.000) (Table 6). In addition, Mann-Whitney U statistical test for pairwise comparison revealed significant differences between the lamellae, tufts and spindle types of the teeth related to the arch and loop fingerprint patterns (*P* value = 0.000), between the lamellae types of the teeth related to the arch and whorl fingerprint patterns (*P* = 0.018), non-significant difference between the tufts types among the teeth related to the arch and whorl fingerprint patterns (*P* = 0.886) and, significant difference between the spindle types among the teeth related to the arch and whorl fingerprint patterns (*P* = 0.000) and between the lamellae, tufts and spindle types of the teeth related to the loop and whorl fingerprint patterns (*P* = 0.000) (Table 7).

**Table 5:** Frequency and percentage of the types of the hypocalcified structures among the teeth related to the arch, loop and whorl fingerprint patterns

Frequency (%)			
Hypocalcified structures	Arch	Loop	Whorl
Type A enamel lamella	70 (71.40)	4 (6.70)	49 (56.40)
Type B enamel lamella	20 (20.40)	6 (10.00)	21 (24.10)
Type C enamel lamella	8 (8.20)	50 (83.30)	17 (19.50)
One-third enamel tuft	149 (64.20)	36 (35.30)	108 (63.50)
One-fifth enamel tuft	83 (35.80)	66 (64.70)	62 (36.50)
Short enamel spindle	246 (69.7)	13 (6.4)	120 (42.5)
Medium enamel spindle	82 (23.2)	36 (17.8)	122 (43.3)
Long enamel spindle	25 (7.1)	153 (75.7)	40 (14.2)

**Table 6:** Independent-samples Kruskal-Wallis statistical test for the results of the types of enamel lamellae, tufts and spindles among the teeth related to the arch, loop and whorl fingerprint patterns

Statistics	Lamellae	Tufts	Spindles
Total N	245	504	837
Test Statistic	94.633^a^	27.377^a^	319.258^a^
Degree Of Freedom	2	2	2
*P *value.(2-sided test)	0.000	0.000	0.000

**Table 7:** Mann-Whitney U statistical test for the results of the types of enamel lamellae, tufts and spindles among the teeth related to the arch and loop fingerprint patterns

Fingerprint	Lamellae			Tufts			Spindles		
	Mann-Whitney U	Z	*P* value. (2-tailed)	Mann-Whitney U	Z	*P* value. (2-tailed)	Mann-Whitney U	Z	*P* value. (2-tailed)
Arch X Loop	560.000	−9.285	0.000	8409.000	−4.891	0.000	7278.500	−16.875	0.000
Arch X Whorl	3533.000	−2.366	0.018	19583.000	−0.143	0.886	36152.000	−6.739	0.000
Loop X Whorl	840.000	−7.556	0.000	6222.000	−4.508	0.000	9582.000	−13.270	0.000

### Micromorphological results

**Fig. 4:**
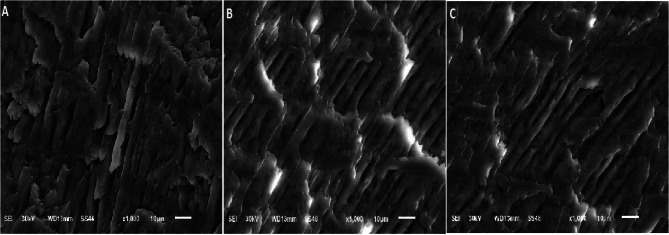
SEM micromorphological investigation of the enamel related to the arch fingerprint patterns (**A**), shows enamel rods of irregular and uneven thickness. The rods are wrapped in a thick layer of rod sheaths that contain higher amounts of the organic matrix. The rods are separated from each other with a thin layer of interrod substance. The enamel related to the loop fingerprint patterns (**B**) shows enamel rods of regular and even thickness. The rods are wrapped in a thin layer of rod sheaths that contains little amounts of the organic matrix. The rods are separated from each other with a thick layer of interrod substance. Whereas, the enamel related to the whorl fingerprint patterns and compared to the enamel related to the arch fingerprint patterns (**C**) shows less or more irregular enamel rods of uneven thickness. The rods are wrapped in less or more a thick layer of rod sheaths that contain higher amounts of the organic matrix. The rods are separated from each other with a less or more thin layer of interrod substance (1000 × magnification)

The rod, the rod sheath, and interdental substances identified enamel. Regarding the specimens related to the arch fingerprint patterns, the micromorphological investigation showed enamel rods of irregular and uneven thickness. The rods were wrapped in a thick layer of rod sheaths that contain higher amounts of the organic matrix. The rods were separated from each other with a thin layer of interrod substance. Specimens related to the loop fingerprint patterns showed enamel rods of regular and even thickness. The rods were wrapped in a thin layer of rod sheaths that contain small amounts of the organic matrix. The rods were separated from each other with a thick layer of interrod substance. Meanwhile, specimens related to the whorl fingerprint patterns and compared to the enamel related to the arch fingerprint patterns showed less or more irregular enamel rods of uneven thickness. The rods were wrapped in less or more a thick layer of rod sheaths that included higher amounts of the organic matrix. The rods were separated from each other with a less or more thin layer of interrod substance (Fig. 4).

### Microchemical results

**Fig. 5:**
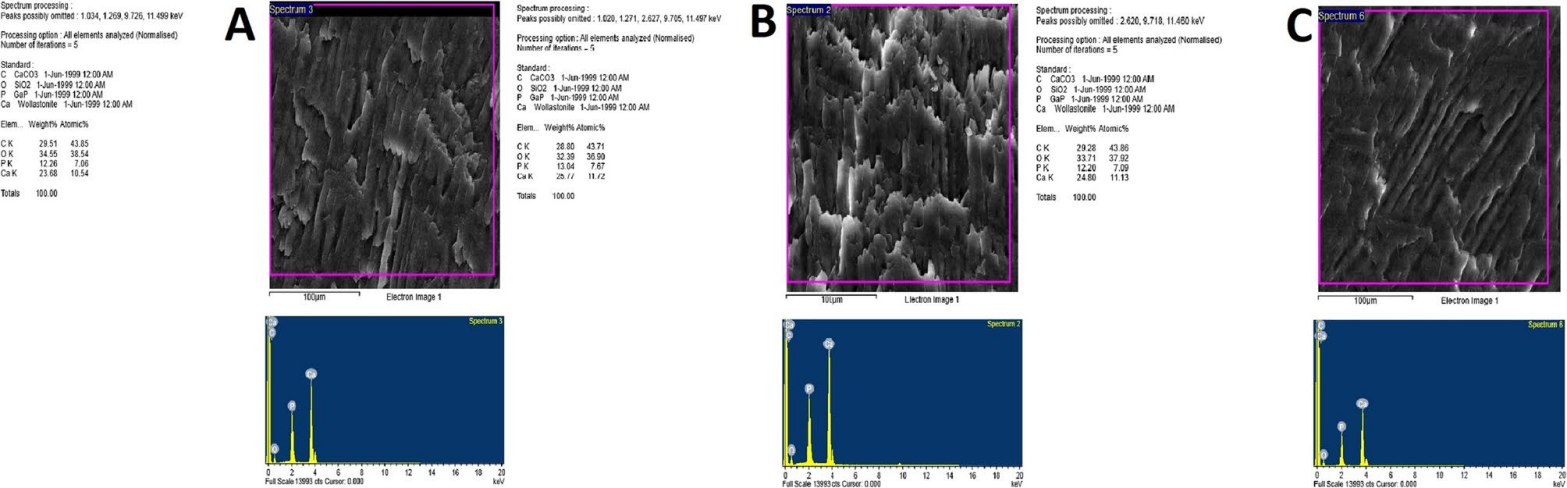
EDX element mapping for the specimens related to the arch fingerprint patterns (**A**), showing a lower amount of Ca and P which indicate lower degree of mineralization. The EDX results show a higher amount of C and O, which indicates a higher amount of enamel organic matrix. The specimens related to the loop fingerprint patterns (**B**) have a higher amount of Ca and P which indicate higher degree of mineralization. The EDX results show lower amount of C and O, which indicates a lower amount of enamel organic matrix. Whereas, specimens related to the whorl fingerprint patterns and compared to the specimens related to the arch fingerprint patterns (**C**) shows less or more lower amount of Ca and P which indicate less or more lower degree of mineralization. The EDX results showing less or more higher amount of C and O which indicate less or more higher amount of enamel organic matrix

The EDX results for the enamel which known to be consists mainly of two compartments inorganic matrix represented by hydroxyapatite crystals resulting on the presence of Ca (kα) and P (kα) and enamel organic matrix represented by non-collagenous proteins resulting on the presence of both carbon (kα) and oxygen (kα) (Fig. 5). Elemental analysis for the Ca weight content (kα) in the enamel of the teeth related to the arch, loop, and whorl fingerprints patterns was 23.53 ± 0.15, 25.72 ± 0.02, and 24.77 ± 0.02, respectively (Table 8). One-way ANOVA statistical test revealed a significant difference for the Ca weight content (kα) between three groups (F ratio = 1517.100, *P* value = 0.000) (Table 9). LSD post-hoc test revealed significant differences for the Ca weight content (kα) between teeth related to the arch and loop, arch and whorl, and between loop and whorl fingerprints patterns (*P* value = 0.000) (Table 9). Regarding P weight content (kα) in the enamel of the teeth related to the arch, loop, and whorl fingerprints patterns, the mean values and SD were 12.20 ± 0.05, 13.02 ± 0.01, and 12.17 ± 0.01, respectively (Table 8). One-way ANOVA statistical test revealed a significant difference for the P weight content (kα) between three groups (F ratio = 2175.255, *P* value = 0.000) (Table 9). LSD post-hoc test revealed significant differences for the P weight content (kα) between teeth related to the arch and loop and between loop and whorl fingerprints patterns (*P* value = 0.000) and non-significant difference for the P weight content (kα) between teeth related to the arch and whorl fingerprints pattern (*P* value = 0.064) (Table 9).

**Table 8:** Descriptive statistics for the Ca, P, C and O weight content (k) among the enamel of the teeth related to the arch, loop and whorl fingerprints patterns

Fingerprint	**Mean ± SD**			
	**Ca**	**P**	**C**	**O**
Arch	23.53 ± 0.15	12.20 ± 0.05	29.47 ± 0.02	34.51 ± 0.01
Loop	25.72 ± 0.02	13.02 ± 0.01	28.77 ± 0.02	32.36 ± 0.02
Whorl	24.77 ± 0.02	12.17 ± 0.01	29.24 ± 0.02	33.68 ± 0.02

**Table 9:** One-way ANOVA and LSD post-hoc statistical tests for Ca, P, C and O weight content (k) among the enamel of the teeth related to the arch, loop and whorl fingerprints patterns

**Fingerprint**	**LSD post-hoc test**							
	**Ca**		*P*		**C**		**O**	
	**Mean Difference**	*P*value	**Mean Difference**	*P*value	**Mean Difference**	*P*value	**Mean Difference**	*P*value
Arch X Loop	−2.19100^*^	0.000	−0.81400^*^	0.000	0.70100*	0.000	2.15200*	0.000
Arch X Whorl	−1.24200^*^	0.000	0.02800	0.064	0.23500*	0.000	0.82500*	0.000
Loop X Whorl	0.94900^*^	0.000	0.84200*	0.000	−0.46600*	0.000	−1.32700*	0.000
One-way ANOVA (F ratio and *P* Value)	(1517.100, 0.000)		(2175.255, 0.000)		(1800.429, 0.000)		(26588.940, 0.000)	

Meanwhile, the elemental analysis for the C weight content (kα) in the enamel of the teeth related to the arch, loop, and whorl fingerprints patterns was 29.47 ± 0.02, 28.77 ± 0.02, and 29.24 ± 0.02, respectively (Table 8). One-way ANOVA statistical test revealed a significant difference for the C weight content (kα) among three groups (F ratio = 1800.429, *P* value = 0.000) (Table 9). LSD post-hoc test revealed significant differences for the C weight content (kα) between teeth related to the arch and loop, arch and whorl, and between loop and whorl fingerprints patterns (*P* value = 0.000) (Table 9). Whereas, the mean values and SD for the O weight content (kα) in the enamel of the teeth related to the arch, loop, and whorl fingerprints patterns were 34.51 ± 0.01, 32.36 ± 0.02, and 33.68 ± 0.02, respectively (Table 8). One-way ANOVA statistical test revealed a significant difference for the O weight content (kα) between three groups (F ratio = 26588.940, *P* value = 0.000) (Table 9). LSD post-hoc test revealed significant differences for the C weight content (kα) between teeth related to the arch and loop, arch and whorl, and between loop and whorl fingerprints patterns (*P* value = 0.000) (Table 9).

## Discussion

Homogeneity ensures boosting the research’s internal validity and the reliability of its outcomes, and the consistency of study participants. Choosing a homogeneous group was dependent on the fact that participants with similar characteristics enable researchers to extract specific information related to their research objectives [30]. Therefore and for consistency, the upper primary 2nd molar was the only tooth selected for recording the ameloglyphics, and for investigating the hypocalcified areas and micromorphological patterns, and for microchemical elemental analyses. In addition, the fingerprint patterns were recorded from the left thumb of the male child or the right thumb of the female child, and this could be attributed to the fact that in the official, legal, and other important matters, the right thumb of females and the left thumb of males are used as an identification tool [31].

Regarding the fingerprint subpatterns, our results comes with Abdel Aziz et al. (2019) who selected 60 adult healthy Egyptians (30 males and 30 females) from the Forensic Medicine and Clinical Toxicology Department in Alexandria University to study the relationship of cheiloscopy and dactylography to ABO blood groups. They found that ulnar loop fingerprint pattern represented the highest percentage among Egyptian males (63.3%) followed by plain whorl (13.5%), central pocket whorl (10%) and plain arch (3.3%), tented arch (3.3%), radial loop (3.3%), and double loop (3.3%). Whereas the Egyptian females showed that ulnar loop fingerprint pattern was 53.3%, plain whorl (23.5%), tented arch (10%), radial loop (6.6%), and double loop (6.6%), central pocket whorl (0%), and plain arch (0%) [32].

In contrast, Zayed et al. (2011) conducted a study to evaluate the impact of gender and race on the system of fingerprint recognition in Egyptians compared to Malaysians. They recruited 200 medical students (100 males and 100 females) at the Forensic Medicine and Clinical Toxicology Department of Cairo University. They reported that the distribution of the ulnar loop fingerprint pattern was the highest (62.2%) followed by arches (24%), whorls (11.5%), and radial loops (2.3%) among the Egyptian medical students. In addition, they found that the percentages between the Egyptian males were 56%, 21.8%, 17.8% and 4.4% for the ulnar loop, whorls, arches and radial loop, respectively and in the females the percentages were 68.4%, 30.2%, 1.2% and 0.2% for the ulnar loop, arches, whorls and radial loop, respectively [33].

Acid etching was performed for the central region of the middle one-third of the buccal surface using 37% orthophosphoric acid for 30 s to record toothprint patterns. Etching was supposed to remove the outer aprismatic layer and expose the underlying prismatic rods. This procedure was dependent on the fact that enamel thickness approximately 0.5–1 mm in primary teeth, and the outer surface layer of enamel appears as a structureless or aprismatic. The aprismatic layer is more frequently seen in more than 60% of the primary teeth with a thickness of 16–45 μm, where the width of this zone in primary teeth is larger, compared to permanent teeth. Therefore, the rod end patterns or toothprints taken from primary teeth were considered as fixed marks, not altered by exposure to adverse conditions, and are unique to each individual. Hence, they could be used as a reliable source for personal identification of the children [34].

Considering toothprint patterns, our results comes with the results reported by Suvarnan et al. (2024), who reported that the following enamel rod end patterns were detected and recorded at the enamel surface of primary anterior teeth, namely wavy branched, linear branched, and unbranched, whorl, loop, and stem-like. They found wavy branched pattern was the only pattern that predominated in all the samples, and only wavy branched and linear branched emerged as a single subpattern. The patterns of linear unbranched, whorl, and stem-like were never observed alone; rather, they were combined with two, three, or four other subpatterns, primarily wavy branched and linear branched [35].

In the present study, the teeth related to the arch fingerprint pattern had a higher percentage of the linear unbranched pattern, the teeth related to the loop fingerprint pattern had a higher percentage of the wavy branched pattern, and the teeth related to the whorl fingerprint pattern had a percentage distributed between wavy branched, linear unbranched, and linear branched. Ameloblasts, as they lay down enamel and traverse to fill the space between the dentin and outer surface with a mineralized structure, create three zones of enamel structure. From inward to outward, they are termed inner enamel zone; almost perpendicular to the DEJ, enamel decussation zone; that follow a multiserial patterning from the DEJ to the outer surface and, enamel parallel prism zone; in which enamel prism bundles align in a more parallel arrangement with a predominant keyhole appearance when viewed in cross Sect [36]. By having a wavy or branching enamel, cracks are less likely to propagate directly through the enamel, instead, the cracks will be redirected and deflected, which increases the enamel’s resistance to fracture [37]. Therefore, the wavy course and branching of the enamel rods contribute to the strength and fracture resistance of enamel.

It was found that an association of moderate correlation between fingerprint and toothprint patterns. This could be attributed to the fact that the fingerprints and toothprints share a common root in their development, as both of them develop during the 3rd week of intrauterine life from the same ectodermal tissue, therefore, they have a potential genetic link. Teeth were considered as hard tissue analog to fingerprints and unique for an individual as fingerprints. In addition, abnormalities in fingerprints and toothprints could be influenced by a combination of hereditary and environmental factors. When the combined factors exceed a certain level, abnormalities are expected to appear. Odontogenesis is genetically modulated, and the formation of enamel is a dynamic process and highly organized. Ameloblasts lay down enamel rods in an intertwining and undulating path, and this process is reflected on the outer surface of the enamel as a series of enamel rod end patterns [38].

The hypocalcified areas including enamel lamellae, tufts and enamel spindles were presented with a greater number in the teeth related to the arch fingerprint patterns and with a fewer number in the in the teeth related loop fingerprint patterns. Hypocalcified areas in enamel are of clinical significance as they can weaken the enamel structure and increase the predisposition of enamel to dental caries. These regions are less mineralized and therefore more permeable, allowing bacteria and acids to penetrate deeper into the tooth structures [39]. The greatest amount of demineralization in enamel caries arises at a subsurface level covered by a surface layer that appears relatively unaffected by the attack, and this means the majority of the mineral loss during initial stages of demineralization occurs at a distance away from the enamel surface [40].

Kelly et al. (2020) stated that the arrangement of enamel rods contributes to the strength and hardness of enamel and the developmental defects in enamel, where variations in rod thickness or alignment could increase the risk of caries [41]. In the present study, the micromorphological results comes with the findings of Roberts et al. (2022) who reported that enamel rod sheaths are considered as a primary pathway for acid attack and demineralization, potentially leading to the formation of tubular carious lesions that can be clinically challenging to diagnose [42].

The integrity and strength of tooth enamel are directly related to the composition of its mineral content, primarily hydroxyapatite. Ca and P are the main building blocks of hydroxyapatite crystals, which provide enamel with its resistance to wear and hardness. C and O are also present in enamel matrix, forming the organic matter that contributes to the overall hardness and supports the mineral structure [43]. The microchemical results comes with the findings of Ersen et al. (2025) who reported that the difference in elemental composition between carious and normal enamel suggests that caries is a process of enamel demineralization, where minerals are lost from the enamel structure. This process weakens the enamel and makes it more susceptible to further damage [44].

The research question of the present study was clear, focusing on addressing the gap between the finger dermatoglyphics and susptability of the primary teeth to dental caries. The research design employed appropriate methods such as recording the finger dermatoglyphics and ameloglyphics and finding the relationship between them. Investigating the hypocalcified areas and micromorphological pattern of the enamel with its microchemical elemental analyses were the tools determining the susptability of these teeth to dental caries.

In coincidence with our points of strength, there are certain limitations such the multifactorial nature of the carious process and choosing the participants from three dental centers of the same governorate. In addition, the finger dermatoglyphics and ameloglyphics were recorded using traditional methods and advanced software for biometric analyses should be considered in the future for identification and comparison. Within the limitations of the current study, we reject the null hypothesis and conclude that fingerprint patterns might be linked to the development of dental caries and can potentially serve as a non-invasive diagnostic tool for identifying individuals at risk. Therefore ongoing researches are needed to provide valuable insights into how new technologies could be used for easy implementation of recording the finger dermatoglyphics and ameloglyphics and correlating them with susceptibility of primary teeth to dental caries.

## Conclusion

The presence of arch fingerprint patterns might be associated with a higher risk of ECC whereas, loop fingerprint patterns might be associated with a lower risk of ECC and whorl fingerprint patterns might be associated with a moderate risk of developing ECC.

## Data Availability

The datasets generated during the current study are available from the corresponding author upon reasonable request.
